# A Phase 1 Dose Escalation of Lapatinib and Paclitaxel in Recurrent Ovarian Cancer

**DOI:** 10.3390/cancers18040626

**Published:** 2026-02-14

**Authors:** Connie D. Cao, Joseph Robert McCorkle, Donglin Yan, Hoda Saghaeiannejad Esfahani, Rani Jayswal, Dava Piecoro, Ning Li, Lauren A. Baldwin, Rachel W. Miller, Christopher P. Desimone, Charles S. Dietrich, Frederick R. Ueland, Jill M. Kolesar

**Affiliations:** 1 Division of Gynecologic Oncology, Department of Obstetrics and Gynecology, University of Kentucky Markey Cancer Center, Lexington, KY 40536, USA; 2 Markey Cancer Center, University of Kentucky, Lexington, KY 40536, USA; 3 Department of Pharmacy Practice and Science, College of Pharmacy, University of Kentucky, Lexington, KY 40506, USA; 4 Department of Pathology & Laboratory Medicine, University of Kentucky Markey Cancer Center, Lexington, KY 40536, USA; 5 Department of Biostatistics, College of Public Health, University of Kentucky, Lexington, KY 40506, USA; 6 Department of Pharmaceutical Sciences and Experimental Therapeutics, College of Pharmacy, University of Iowa, 180 S Grand Ave, 310L CPB, Iowa City, IA 52242, USA

**Keywords:** lapatinib, paclitaxel, recurrent ovarian cancer

## Abstract

Objective: The development of ABCB1-mediated resistance limits the clinical efficacy of paclitaxel. Lapatinib is a small-molecule reversible tyrosine kinase and ABCB1 inhibitor that could prevent resistance. Our objective was to determine a recommended phase 2 dose (RP2D) of the combination of paclitaxel and lapatinib. Methods: A phase 1 dose-escalation study utilizing a Bayesian optimal interval (BOIN) design in recurrent ovarian cancer patients was conducted. Patients were pretreated with pulsed lapatinib in the 48 h preceding weekly paclitaxel (80 mg/m^2^) in 28-day cycles for up to three cycles. We evaluated three lapatinib doses, escalating from 750 to 2000 mg orally twice daily. Results: Sixteen patients were eligible and evaluable for efficacy and toxicity. Patients received a median of three prior therapies. Three patients were treated at dose level 1, six at dose level 2, and seven at dose level 3. There was one dose-limiting toxicity (DLT) in dose level 2 (diarrhea) and another in dose level 3 (neutropenia), with a posterior DLT estimate of 0.17, 95% credible interval of (0.01, 0.53) for dose level 3 based on isotonic regression. The most common grade 1–2 adverse effects were diarrhea (87.5%), leukopenia (56.3%), and anemia (50%). One (6.25%) patient had a complete response, and seven (43.75%) patients had partial responses for an overall response rate (ORR) of 50%. The clinical responses are supported by a significant decreasing trend in CA 125 over six cycles (*p* = 0.0001). Among the seven patients treated at the RP2D, the ORR was 71.4%. Conclusions: The combination of paclitaxel and lapatinib was safe and demonstrated an efficacy signal. The RP2D was weekly paclitaxel 80 mg/m^2^ combined with lapatinib 2000 mg twice daily two days before the paclitaxel dose. This trial was registered at ClinicalTrials.gov ID: NCT04608409.

## Simple Summary

Platinum-resistant ovarian cancer holds a poor prognosis and is often treated with single-agent chemotherapy. The development of ABCB1-mediated resistance limits the clinical efficacy of paclitaxel in recurrent ovarian cancer. Lapatinib is a small-molecule reversible tyrosine kinase and ABCB1 inhibitor that has been shown to reverse paclitaxel resistance during in vitro studies. The combination of pulsed lapatinib 2000 mg twice daily given two days prior to weekly paclitaxel 80 mg/m^2^ has a clinical efficacy signal and is safe in recurrent ovarian cancer patients.

## 1. Introduction

In 2025, 20,890 new cases of ovarian cancer and 12,730 deaths have been estimated. Ovarian cancer encompasses histologically similar tumors that arise from the epithelial tissue of the ovary, fallopian tube, or peritoneum and is the sixth leading cause of cancer-related deaths in females [1]. Most women have been diagnosed with advanced-stage disease with a 5-year survival rate of approximately 50% with minimal improvement in survival outcomes in the past decade [2].

Initial therapy for ovarian cancer includes cytoreductive surgery and platinum-based chemotherapy. However, the majority of patients will recur and become platinum-resistant, defined as recurrence within six months of last platinum chemotherapy. The treatment for platinum-resistant ovarian cancer often consists of a nonplatinum agent (e.g., weekly paclitaxel, pegylated liposomal doxorubicin, gemcitabine, or topotecan) administered either as a single agent or in combination with bevacizumab [3]. In recent phase 3 trials, objective response rates to single-agent nonplatinum chemotherapy in patients with platinum-resistant ovarian cancer ranged from 4 to 13% [4,5,6]. The addition of bevacizumab, as compared with chemotherapy alone, significantly improved the response rate (27.3% vs. 11.8%) but without benefit in overall survival [7]. Chemotherapy-associated adverse effects, including gastrointestinal toxicity, hematological toxicity, and cumulative neuropathy, limit continued administration and contribute to the observed poor survival. Therefore, new treatment strategies are needed for recurrent ovarian cancer.

Lapatinib is a small-molecule reversible tyrosine kinase inhibitor that targets epidermal growth factor receptor (EGFR) and human epidermal growth factor receptor-2 (HER2). Lapatinib is approved for continuous oral dosing at 1000–1250 mg daily in combination with either an aromatase inhibitor, trastuzumab, or capecitabine in HER2-positive recurrent unresectable breast cancer [8,9,10,11]. Given that many tumors express EGFR and/or HER2, high-dose pulsed lapatinib has been evaluated in advanced solid tumors in a phase 1 dose escalation study of lapatinib administered as a two-day pulse prior to weekly nab-paclitaxel, demonstrating a maximum tolerated dose of 5250 mg/day and a recommended phase 2 dose (RP2D) of 3750 mg/day in divided doses. Dose-limiting toxicities (DLTs) included grade 3 vomiting and grade 4 neutropenia. Among 23 evaluable patients, five had a partial response, and another ten experienced stable disease, suggesting the regimen was tolerable with a preliminary efficacy signal [12].

ABCB1, an ATP-binding cassette subfamily B, member 1, also known as P-glycoprotein (P-gp) or multidrug resistance protein 1 (MDR1), protects normal cells from xenobiotics and other toxic substances [13]. However, in cancer cells, overexpression of ABCB1 can result in drug resistance, especially to taxanes. Paclitaxel administration results in ABCB1 upregulation, inducing its own resistance. ABCB1 overexpression is associated with poor response to taxanes and unfavorable clinical outcomes for patients with ovarian cancer [14].

In addition to inhibiting EGFR and HER2, ABCB1 inhibition is an off-target activity of lapatinib [15,16]. Adding lapatinib to paclitaxel in paclitaxel-resistant ovarian cancer cell lines overcame taxane resistance via inhibition of ABCB1 [16]. Given the doses of lapatinib required to inhibit ABCB1 can cause significant toxicity, we explore a pulsed high dose of lapatinib before and after paclitaxel administration to inhibit ABCB1 during the window of paclitaxel exposure while also minimizing adverse effects. We hypothesized that the combination of weekly paclitaxel and lapatinib could prevent ABCB1 overexpression and the development of taxane resistance; therefore, we performed a phase 1 dose escalation study of the combination of intermittent lapatinib and weekly paclitaxel in recurrent ovarian cancer patients.

## 2. Patients and Methods

### 2.1. Study Design

This study was an open-label, single-center, single-arm, investigator-initiated phase 1 dose escalation study of the safety of lapatinib with weekly paclitaxel in patients with recurrent ovarian cancer. This trial was registered at ClinicalTrials.gov ID: NCT04608409. The study was conducted using a Bayesian optimal interval (BOIN) design. Patients were enrolled in cohorts of three with brief suspension in enrollment on the trial for evaluation of DLTs. Once DLT evaluation was complete for a cohort of patients, the next dose level was determined by available DLT information from all evaluable patients at that dose level based on the BOIN dose escalation/de-escalation rule. The study protocol and modifications were approved by the University of Kentucky Institutional Review Board MCC-20-GYN-06-PMC 8/12/2020 and were conducted in accordance with the Declaration of Helsinki, Good Clinical Practice, and all local and federal regulatory guidelines.

The primary objective of this study was to determine the RP2D of the combination of lapatinib with weekly paclitaxel. Secondary objectives included determining adverse events of the combination of lapatinib and paclitaxel, assessing the plasma concentration of lapatinib, and assessing the proportion of patients with clinical benefit defined as clinical progression-free survival (PFS) at one year from the start of study therapy using radiographic and CA 125 values.

### 2.2. Patient Population

Patients with histologically or cytologically confirmed ovarian cancer who recurred within 12 months after platinum-based chemotherapy, age ≥ 18 years, ECOG performance status ≤ 2, and with adequate organ and marrow function at baseline were eligible for enrollment. Patients with prior or concurrent malignancy whose natural history did not have the potential to interfere with the safety or efficacy of the investigational regimen were also eligible. Patients with a history of uncontrolled intercurrent illness or hypersensitivity to either lapatinib or paclitaxel were ineligible for enrollment. Additionally, patients with malabsorption syndrome, left ventricular ejection fraction < 50%, active HIV, hepatitis B, and hepatitis C with detectable viral load, and patients with baseline > grade 1 neuropathy were not eligible. Patients on medications that are strong inhibitors or inducers of CYP 450 3A4 were ineligible unless they transitioned off these prior to study initiation.

### 2.3. Treatment Plan

The cycle length was 28 days. On days 1, 8, and 15, patients received a fixed dose of paclitaxel 80 mg/m^2^ intravenously. On days 6–7 and 13–14, oral lapatinib was self-administered twice a day. At dose level 1, lapatinib 750 mg was administered twice daily and was escalated with three patients per cohort to dose level 2, lapatinib 1500 mg twice daily, and dose level 3, lapatinib 2000 mg twice daily. After the first cycle, patients continued the combination of lapatinib and paclitaxel for an additional two cycles. Patients could then continue weekly paclitaxel as a single agent.

### 2.4. Assessments

All patients who received at least one dose of study treatment were evaluated for toxicity. Toxicity was graded by the Common Terminology Criteria for Adverse Events version 5. DLT was assessed after the first cycle of treatment. Patients who completed 75% of doses were evaluable for DLTs. DLT was defined by grade 3 febrile neutropenia; grade 4 neutropenia; grade 4 thrombocytopenia; grade 4 anemia not explained by underlying disease; ≥grade 3 nausea, vomiting, or diarrhea that persisted >72 h despite optimal supportive management; or any ≥grade 3 or ≥grade 2 adverse events not improved after stopping the drug combination. The response was assessed by the treating physician in patients who received at least one cycle of treatment by standard-of-care clinical assessments, which were performed after three cycles of a combination of lapatinib and paclitaxel. Blood samples were collected on days 1, 8, and 15 of each cycle for analysis of lapatinib concentrations and ABCB1 expression using cell-free RNA.

### 2.5. Statistical Methods

DLT rate was calculated as the total number of patients experiencing DLTs at the current dose level divided by the total number of patients treated at the current dose level. A target DLT rate was set to be 30% for the study. Dose escalation and de-escalation decisions were determined by all evaluable patients at the current dose level using the BOIN dose escalation/de-escalation rule. The study was planned to enroll a maximum of 15 patients. The RP2D was determined by isotonic regression to pool information across all dose levels.

Patient characteristics, antitumor activity of the drug combination, and safety and tolerability of lapatinib were summarized using descriptive statistics. The clinical benefit rate was defined as the proportion of patients with clinical progression-free survival (PFS) at one year defined as the time from start of the protocol to the time of progression of disease or death, whichever occurred first.

Changes in lapatinib levels from baseline throughout the treatment period were represented by longitudinal profiles and analyzed by a mixed-effects model. Changes in ABCB1 RNA expression were compared between pre- and post-paclitaxel administration.

### 2.6. Plasma Drug Concentrations

Plasma lapatinib concentrations were analyzed among samples from patients who completed dosing for the assigned treatment arm and had blood drawn within 24 h of the last lapatinib dose. The concentration of lapatinib was measured in plasma samples using a 1260 Infinity II LC system interfaced with an Ultivo triple quadrupole mass spectrometer (LC/MSMS) (Agilent Technologies, Santa Clara, CA, USA). Stock solutions of lapatinib (1 mg/mL) and lapatinib-d7 (1 mg/mL) were prepared in methanol. Assay calibration was performed for lapatinib using solutions prepared in blank human peripheral blood plasma (StemCell Technologies, Vancouver, BC, Canada) at concentrations ranging from 25 to 10,000 ng/mL, and calibration curves were linear (R^2^ > 0.999). The lower limit of quantitation was 100 ng/mL, and the upper limit of quantitation was 10,000 ng/mL. Quality controls of 200 and 5000 ng/mL were included with each run.

Each patient specimen, standard, and quality control sample was combined with three parts acetonitrile containing lapatinib-d7 (50 ng/mL). The tubes were vortexed for 0.5 min and then centrifuged for 10 min at 15,000× *g*. Supernatants were transferred to multi-well plates and dried under a stream of nitrogen gas at 40 °C. The dried samples were resuspended in a 1:1 mixture of 10 mM ammonium acetate (pH 4.5) + 0.1% formic acid and acetonitrile + 0.1% formic acid, placed on an orbital shaker for 10 min at 750 rpm, and then loaded into the autosampler.

Liquid chromatography was performed using an (Agilent, Santa Clara, CA, USA) InfinityLab Poroshell 120 PFP column (50 mm × 2.1 mm, 2.7 μm) equipped with a guard column. The mobile phase consisted of (A) 10 mM ammonium acetate (pH 4.5) + 0.1% formic acid and (B) acetonitrile + 0.1% formic acid delivered at 0.3 mL/minute as a linear gradient as follows: 0–2.5 min, 30% to 95% B; 2.5–3.5 min, 95% to 100% B; 3.5–4.0 min, 100% B; 4.0–4.5 min, 100% to 30% B. A 5 min re-equilibration occurred before the injection of the next sample. Electrospray ionization was operated in positive mode (ESI+) with nitrogen as both curtain and collision gas. The Ultivo instrument (Agilent, Santa Clara, CA, USA) parameters were drying gas temperature 250 °C, drying gas flow 11 L/minute, sheath gas temperature 400 °C, sheath gas flow 11.0 L/minute, and nebulizer 50 psi. MRM detected the transitions of lapatinib at *m*/*z* 581.1 → 365.1 (fragmentor voltage 190 V; collision energy 41 V). Data was analyzed using MassHunter Workstation (Agilent Technologies, Santa Clara, CA, USA) software 7700e.

### 2.7. Plasma CA125 Concentrations

Patients’ blood was drawn and processed at the University of Kentucky HealthCare Laboratory to determine serum CA125 concentrations.

## 3. Results

### 3.1. Patient Characteristics

**Figure 1:**
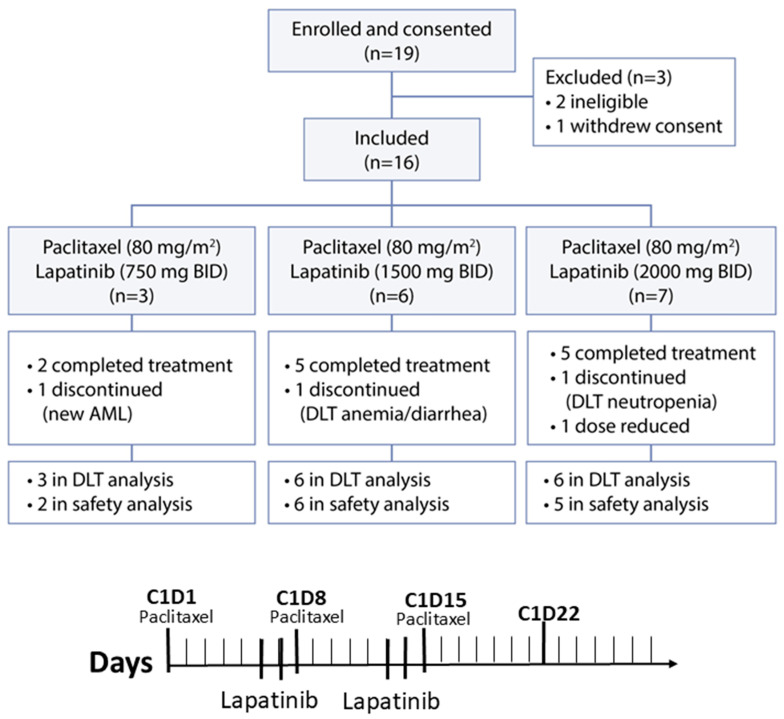
Trial diagram and treatment schedule for a 28-day cycle where on days 1, 8, and 15 patients receive fixed dose paclitaxel 80 mg/m^2^ IV and days 6–7 and 13–14 oral lapatinib self-administered twice daily.

Between March 2021 and May 2023, 19 women with ovarian cancer were enrolled on the study. Two patients were subsequently determined to be ineligible: the first patient had leukocytosis and anemia, and the second patient developed uncontrolled intercurrent infection and leukocytosis prior to starting study therapy. A third patient withdrew consent from the study (Figure 1). In total, 16 patients were evaluable for efficacy, toxicity, and response. The median age was 66 and all patients enrolled were white, 15 subjects had high-grade serous histology, and one had endometrioid histology. The median number of prior lines of systemic therapy was three (range 1–7). The median platinum-free interval for this cohort was 4 months (range 1–9 months). A total of 16 patients received paclitaxel in the primary setting, and seven received prior bevacizumab (Supplementary Table S1).

### 3.2. Safety and Tolerability

The majority of adverse events in all patients receiving lapatinib and paclitaxel were mild to moderate in severity (Table 1 and Supplementary Table S2). Diarrhea (n = 14, 87.5%) was the most common adverse event. Other non-hematological toxicities included hypokalemia (n = 9, 56.3%), hypocalcemia (n = 8, 50%), and hyponatremia (n = 8, 50%). The grade 3/4 non-hematological toxicities included diarrhea (n = 3, 18.8%), hypokalemia (n = 2, 12.5%), acute kidney injury (n = 2, 12.5%), and hyponatremia (n = 1, 6.3%). The most common hematological toxicities were leukopenia (n = 9; 56.3%) and anemia (n = 8; 50%). One patient with a history of prior PARP inhibitor use went off the study after developing a secondary malignancy, which was attributed to prior PARP therapy and determined to be unrelated to the study treatment.

There were two DLTs observed, one for diarrhea and one for neutropenia. One DLT was observed in a subject on dose level 2, and one DLT occurred in a subject treated on dose level 3, with a posterior DLT estimate of 0.17, 95% credible interval of (0.01, 0.53) for dose level 3. Therefore, the RP2D is 80 mg/m^2^ of weekly paclitaxel combined with 2000 mg bid of lapatinib administered two days before the paclitaxel dose. The estimated posterior DLT rate yielded a credible interval of (0.01, 0.53) for both dose level 2 and dose level 3. Following isotonic regression analysis of pooled data from 15 patients across all dose levels, dose level 3 was identified as the recommended maximum tolerated dose (MTD).

### 3.3. Efficacy

**Figure 2:**
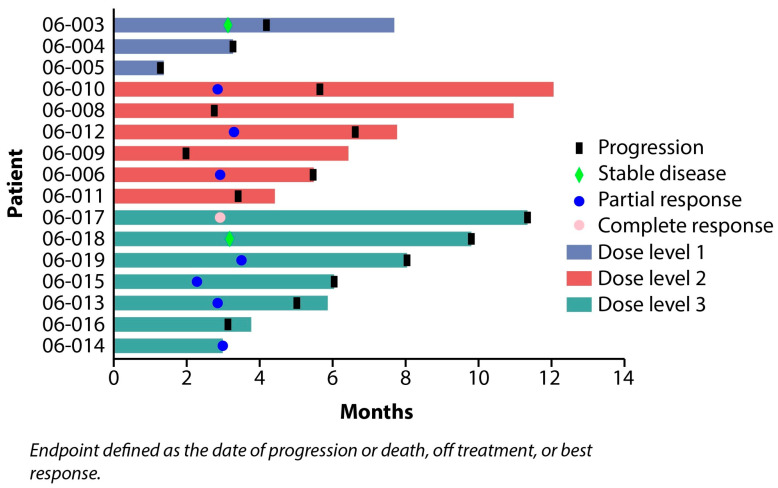
Best responses by dose level.

**Figure 3:**
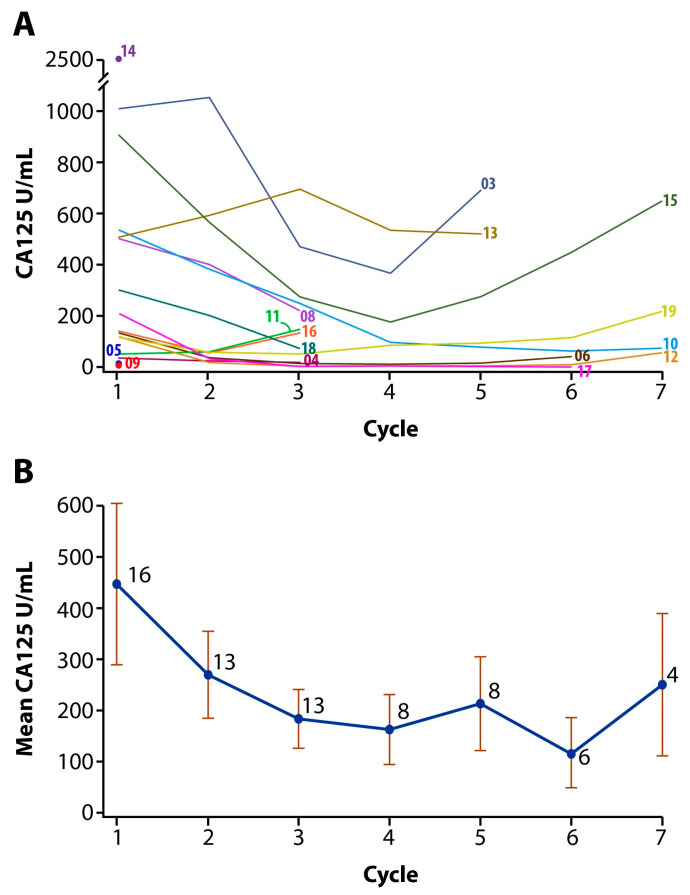
CA125 trend with each treatment cycle separated by individual patient (numbers represent last two digits of patient number) (**A**). Cohort average (bars indicate standard error) (**B**).

**Figure 4:**
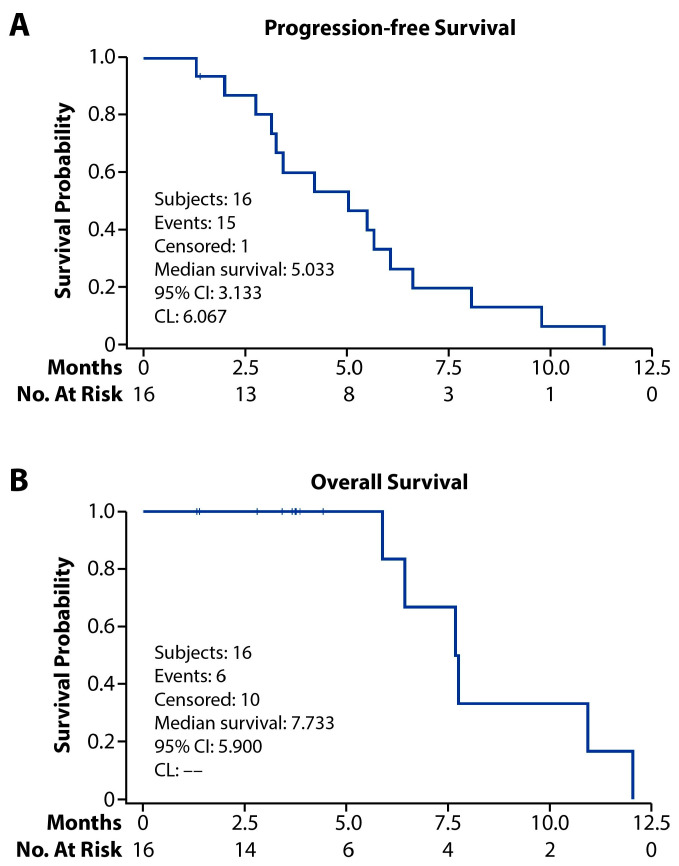
Kaplan-Meier survival plots (**A**). Overall survival (**B**).

**Figure 5:**
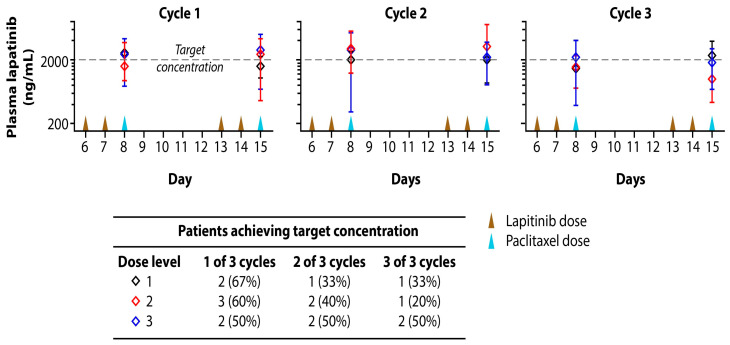
Plasma lapatinib concentrations among enrolled subjects. Concentrations were measured prior to paclitaxel administration following 2 days of BID dosing during Cycles 1–3. The target lapatinib plasma concentration at the time of paclitaxel administration (day 8 and day 15) was 2000 ng/mL (dashed line). Mean values (diamonds) ± standard deviation for each dose level is shown. Timing of lapatinib (brown arrows) and paclitaxel (blue arrows) doses are depicted along the *x*-axis. Numbers of patients reaching target plasma concentration in at least 1, 2 or all 3 cycles are summarized in the adjoining table.

The median number of cycles of paclitaxel and lapatinib was three (range 1–3), and the median total cycles given, including weekly paclitaxel alone, was 5.5 (range 1–8). At the time of data cut-off, all 16 patients were off treatment. One (6.25%) patient had a complete response (CR) and seven (43.75%) had partial responses (PR) for an overall response rate (ORR) of 50%. Two (12.5%) had stable disease (SD) (Figure 2). At dose level 1, only one patient had SD, while three patients achieved a PR at dose level 2. Among the seven patients treated at dose level 3, one patient had a CR, four experienced a PR, and one had a SD for an ORR of 71.4%. Of 16 patients, one patient had endometrioid histology and 15 patients with high-grade serous histology; however, all responses (n = 10) were reported among patients with high-grade serous histology (Supplementary Figure S1). These responses were supported by a significant decreasing trend in CA 125 values over six cycles (*p* = 0.0001) (Figure 3). In total, 15 progression events occurred at the time of data cut-off, and the median PFS was 5.0 months (95% CI: 3.13–6.07) (Figure 4). Six patient deaths were recorded, and the median OS was 7.7 months (95% CI: 5.9-NE) (Figure 5).

### 3.4. Plasma Lapatinib Concentrations

Twice daily lapatinib dosing on days 6, 7, 13, and 14 aimed to achieve a therapeutic concentration of 2000 ng/mL at the time of paclitaxel dosing. Plasma lapatinib concentrations were evaluated in patients on days 8 (192 h) and 15 (360 h) of each treatment cycle, prior to paclitaxel administration, using a validated LC/MS assay (Figure 5; Supplementary Table S3). The plasma concentrations were highly variable with ranges of 1439–2579 ng/mL for dose level 1, 1031–3393 ng/mL for dose level 2, and 1824–2951 ng/mL for dose level 3. No statistically significant intercycle or intracycle differences were observed.

Plasma lapatinib concentrations reached 2000 ng/mL in at least one cycle for 67% of patients in dose level 1, 60% in dose level 2, and 50% in dose level 3. The target threshold was achieved in all three cycles for 33% of patients in dose level 1, 20% in dose level 2, and 50% in dose level 3. Patients were categorized as achieving lapatinib plasma concentrations greater or less than 2000 ng/mL. Among six patients with lapatinib concentrations less than 2000 ng/mL the ORR was 67% (1 CR, 3 PR) and all reported best responses (1 CR, 2 SD, and 3 PR). Among 10 patients who had lapatinib plasma concentration greater than 2000 ng/mL, four patients achieved a PR for an ORR of 40% (Supplementary Figure S2).

## 4. Discussion

This was the first study to report the combination of weekly paclitaxel with pulsed lapatinib in ovarian cancer. The RP2D is 80 mg/m^2^ of weekly paclitaxel combined with 2000 mg twice daily of lapatinib given as a pulsed pretreatment two days before the paclitaxel dose. This was the maximum dose planned for this study due to pill burden and anticipated toxicity. This dose was lower than a prior phase 1 clinical study conducted in patients with advanced solid tumors, which defined the MTD of lapatinib as 2625 mg twice daily dosing as a two-day pulse prior to weekly infusion of nab-paclitaxel, and which demonstrated a grade 3–4 rate of febrile neutropenia of 32%. This prior phase 1 study included up to six dose levels, with the highest lapatinib dose of 7500 mg daily. The majority of the patients included had lung (9/25), esophageal (3/25), and prostate cancers (3/25), as well as one ovarian cancer patient. The grading of diarrhea was in the setting of maximal supportive antidiarrheal medication [12].

We observed two DLTs, one for diarrhea and one for neutropenia, which was similar to the prior study where dose-limiting toxicities were vomiting and neutropenia. The prior study also reported a higher incidence of grade 1–2 diarrhea than continuous dosing schedules of lapatinib that was manageable with antidiarrheal medication [12]. In our study, any grade diarrhea was the most common AE at 87.5%, which was manageable with antidiarrheals. Other studies have reported diarrhea rates as high as 83–100% [17,18]. The incidence of rash with lapatinib ranges from 25 to 64% and was typically grade 1 or grade 2, which either resolved without treatment or improved with topical steroid use [12,18,19]. In our study, three (18.8%) patients reported maculopapular rash. Neuropathy was common and grade 2–3 neutropenia occurred in 4–23% of patients receiving weekly paclitaxel [20,21]. Our study findings of leukopenia were consistent with expected effects of weekly paclitaxel.

Though prior phase 2 studies showed minimal activity of single-agent lapatinib in recurrent ovarian cancer [22,23], there was encouraging anticancer activity of the combination in this study. Among the seven patients treated at RP2D, one patient had a CR, four experienced a PR, and one had SD for an ORR of 71.4%. Despite patients receiving only three cycles of lapatinib in combination with paclitaxel, these results were an improvement from the expected ORR of 20.9% with single-agent weekly paclitaxel in platinum-resistant ovarian cancer patients [20]. In addition, the study population was heavily pre-treated, receiving a median of three prior lines of systemic therapy. Further study, potentially in a phase 2 clinical trial with paclitaxel as a comparator and continuation of lapatinib past 3 cycles, is warranted [23].

Recently the development of the antibody–drug conjugate mirvetuximab soravantansine has altered the treatment paradigms in platinum-resistant ovarian cancer for those with tumors who are folate receptor α positive. The MIRASOL study demonstrated improved overall survival with mirvetuximab than chemotherapy (median 16.46 months vs. 12.75 months; HR for death 0.67; 95% CI (0.50–0.89)) in patients who are folate receptor α positive [24]. In the earlier SORAYA trial, over half of the patients screened were found to be folate receptor α negative and ineligible for treatment, demonstrating the ongoing need for treatments in ovarian cancer patients without targetable mutations [25].

The target therapeutic concentration of lapatinib of 2000 ng/mL at time of paclitaxel dosing was achieved in all three cycles for 33% of patients in dose level 1, 20% in dose level 2, and 50% in dose level 3, suggesting a lack of a linear dose response with pulsed dosing. Plasma concentration did not predict response, likely related to plasma concentrations not being reflective of tumor concentrations. The ORR was 67% among subjects with lapatinib concentrations less than 2000 ng/mL compared to 40% for those with plasma concentrations greater than 2000 ng/mL. The lack of prognostic power was likely influenced by the high degree of variability observed in plasma lapatinib concentrations. A number of factors may have contributed to this variability, including small sample size, variable plasma sampling times, and dose reductions.

Strengths of the study include the BOIN design, enrollment of patients with predominantly high-grade serous ovarian cancer histology, long-term follow up data, and pharmacokinetic analysis to assess whether therapeutic concentrations of lapatinib were achieved. Limitations of this study included small cohort size, which limited the precision of the DLT estimation, but as is common with phase 1 studies, recruitment at a single institution, and limited patient diversity that may limit generalizability of the findings. An additional limitation of this study was the treating physician who assessed efficacy, although this is common in phase 1 trials where efficacy is not the primary endpoint. In addition, patients only received three cycles of lapatinib in combination with weekly paclitaxel, limiting assessment of outcomes if lapatinib were continued.

## 5. Conclusions

In conclusion, the combination of weekly paclitaxel with high-dose pulsed lapatinib demonstrated encouraging clinical anticancer activity in heavily pre-treated, recurrent ovarian cancer patients. This combination warrants further exploration in ovarian cancer.

## Figures and Tables

**Table 1:** Adverse events grade 3/4 versus any grade.

	TOTAL N = 16	
**Toxicity N (%)**	**GRADE 3/4**	**ANY GRADE**
**Hematological toxicity**		
Anemia	1 (6.3)	8 (50.0)
Thrombocytopenia	1 (6.3)	3 (18.8)
Leukopenia	1 (6.3)	9 (56.3)
**Non-hematological toxicity**		
Alanine aminotransferase increased	0 (0)	3 (18.8)
Aspartate aminotransferase increased	0 (0)	4 (25.0)
Creatinine increased	2 (12.5)	6 (37.5)
Abdominal pain	0 (0)	2 (12.5)
Constipation	0 (0)	2 (12.5)
Diarrhea	3 (18.8)	14 (87.5)
Nausea	0 (0)	4 (25.0)
Fatigue	0 (0)	3 (18.8)
Hyperkalemia	0 (0)	4 (25.0)
Hyperphosphatemia	0 (0)	2 (12.5)
Hypercalcemia	0 (0)	4 (25.0)
Hyponatremia	1 (6.3)	8 (50.0)
Hypokalemia	2 (12.5)	9 (56.3)
Hypophosphatemia	0 (0.0)	3 (18.8)
Hypocalcemia	0 (0)	8 (50.0)
Hypomagnesemia	0 (0)	7 (43.8)
Hypoalbuminemia	0 (0)	4 (25.0)
Back pain	0 (0)	3 (18.8)
Muscle cramp	0 (0)	2 (12.5)
Zeripheral sensory neuropathy	0 (0)	2 (12.5)
Dyspnea	0 (0)	4 (25.0)
Alopecia	0 (0)	2 (12.5)
Rash maculo-papular	0 (0)	3 (18.8)

Note: A subject is counted only once within a toxicity category. The toxicity with the highest grade is chosen when there are multiple toxicities with different grades for one subject. All percentages are based on the number of subjects in the safe population.

## Data Availability

The data presented in this study are available on request from the corresponding author due to informed consent restrictions.

## Supplementary Materials

The following supporting information can be downloaded at: https://www.mdpi.com/article/10.3390/cancers18040626/s1, Figure S1: Best Responses by Histology; Figure S2: Best Responses by Plasma Concentration; Table S1: Patient demographics; Table S2: Adverse Events Grade 3/4 versus Any Grade; Table S3: Plasma lapatinib C_ss_.

## Abbreviations

The following abbreviations are used in this manuscript:

SD	Stable Disease
BID	Twice a day
ORR	Overall Response Rate
PR	Partial Response
CR	Complete Response
DLT	Drug Limiting Toxicity
