# Supplementary material for: A Phase 1 Dose Escalation of Lapatinib and Paclitaxel in Recurrent Ovarian Cancer
Source: Cancers (Basel). 2026 Feb 14;18(4):626. doi: 10.3390/cancers18040626 (PMC12939439; doi:10.3390/cancers18040626)
Supplement: Supplementary file 1 [file cancers-18-00626-s001.zip › cancers-4123808-supplementary.pdf]

Supplementary Figure S1. Best Responses by Histology

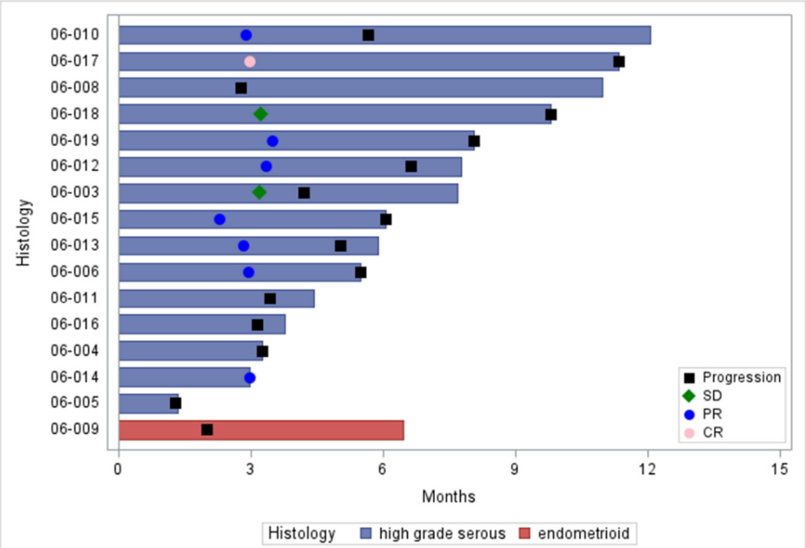

*Best response: SD="stable disease", PR="partial response", CR="complete response"
*Endpoint defined as the date of progression,death, off treatment, or best response

Supplementary Figure S2. Best Responses by Plasma Concentration

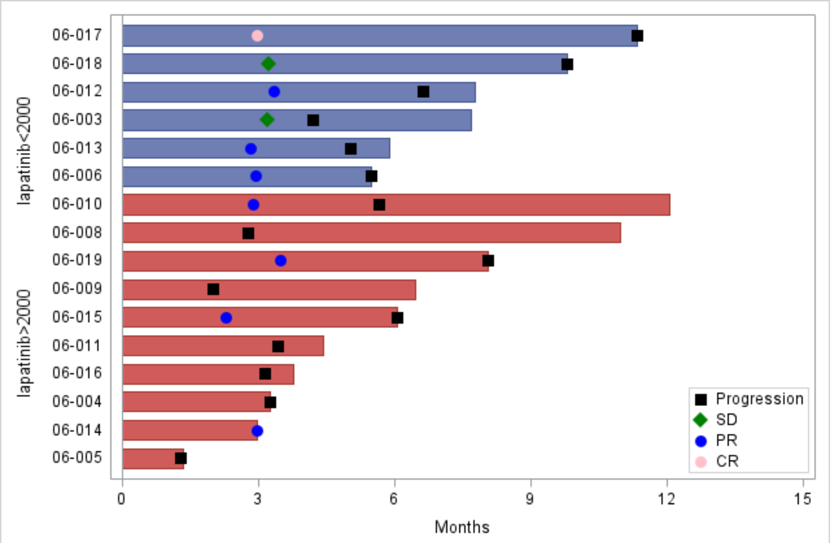

*Best response: SD="stable disease", PR="partial response", CR="complete response"
*lapatinib unit (ng/mL)
*Endpoint defined as the date of progression,death, off treatment, or best response

Supplementary Table S1. Patient demographics

**Characteristic**	**Dose level 1** n=3	**Dose level 2** n=6	**Dose level 3** n=7	**All patients** n=16
Median Age	75	64	66	67.5
White, non-Hispanic	3	6	7	16
ECOG				
0	0	1	4	5
1	3	4	3	7
2	0	1	0	1
Histologic Subtype				
Serous	3	5	7	15
Endometrioid	0	1	0	1
Prior Paclitaxel				
Yes	3	6	7	16
No	0	0	0	0
Prior Bevacizumab				
Yes	1	1	5	7
No	2	5	2	9
Median Prior Lines Therapy (range)				
	1 (1-5)	2 (1-7)	4 (2-6)	3 (1-7)

Supplementary Table S2. Adverse Events Grade 3/4 versus Any Grade

	**TOTAL N= 16**	
**Toxicity N (%)**	**GRADE 3 / 4**	**ANY GRADE**
**Non-hematological toxicity**		
Blood bilirubin increased	0 (0)	2 (12.5)
Alkaline phosphatase increased	0 (0)	3 (18.8)
Hyperglycemia	0 (0)	4 (25.0)
Fever	0 (0)	2 (12.5)
Upper respiratory infection	0 (0)	2 (12.5)
Cough	0 (0)	2 (12.5)
Hoarseness	0 (0)	2 (12.5)

Supplementary Table S3. Plasma lapatinib C_{ss}

Dose level	n	Cycle	Time (day)	C_{ss} (ng/mL)
1 (750 mg BID)	3	1	8	2579 (± 1620)
	2	1	15	1630 (± 590)
	2	2	8	2002 (± 750)
	2	2	15	2002 (± 1120)
	1	3	8	1439
	2	3	15	2422 (± 1460)
2 (1500 mg BID)	4	1	8	1593 (± 2100)
	4	1	15	2370 (± 1910)
	3	2	8	2987 (± 2900)
	3	2	15	3393 (± 3940)
	2	3	8	1499 (± 790)
	3	3	15	1031 (± 600)
3 (2000 mg BID)	3	1	8	2490 (± 1720)
	4	1	15	2851 (± 2160)
	4	2	8	2951 (± 2650)
	4	2	15	2312 (± 1490)
	4	3	8	2175 (± 1790)
	4	3	15	1824 (± 1130)

Data are expressed as mean ± SD
